# Le profil des urgences urologiques dans un hôpital régional au Sénégal: étude rétrospective de 20 mois

**DOI:** 10.11604/pamj.2022.42.302.34654

**Published:** 2022-08-22

**Authors:** Thierno Oumar Diallo, Ibrahima Diabaté, Mamadou 2 Barry, Oumar Raphiou Bah

**Affiliations:** 1 Service d´Urologie, Hôpital Régional de Kolda, Kolda, Sénégal,; 2 Service d´Urologie de l´Hôpital National Ignace Deen, Université de Conakry, Conakry, Guinée,; 3 Service d´Urologie, Hôpital Régional de Louga, Louga, Sénégal

**Keywords:** Urgences urologiques, rétention d’urine vésicale, infections urogénitales, cathétérisme sus-pubien, colique néphrétique, Urological emergencies, urinary retention, urogenital infections, suprapubic catheterization, renal colic

## Abstract

**Introduction:**

les urgences urologiques sont diverses et variées, et sont fréquemment rencontrées en pratique urologique. L´objectif de ce travail était d´étudier les aspects épidémiologiques et la prise en charge des urgences urologiques au centre hospitalier régional de Kolda, Sénégal.

**Méthodes:**

nous avons réalisé une étude rétrospective et descriptive d´une durée de 20 mois (janvier 2020 et le 31 août 2021) colligeant les dossiers de tous les patients reçus pour une urgence urologique.

**Résultats:**

les urgences urologiques représentaient 3,6% des patients admis au Service d´Accueil des Urgences (SAU) et 20,4% des consultations urologiques. L´âge moyen des patients était de 51,9 ± 21,5 ans avec des extrêmes de 1 an et de 98 ans. La rétention d´urine vésicale a été l´urgence urologique la plus fréquente, observée dans 249 cas (57,3%), suivie des infections urogénitales 89 (20,5%). La colique néphrétique avait concerné 48 (11%) des patients. Pour la prise en charge des urgences, le sondage vésical a été réalisé chez 193 patients (44,3%). Les actes chirurgicaux les plus fréquents étaient le cathétérisme sus-pubien 42 (51,9%) et l´exploration scrotale en urgence 15 (18,6%).

**Conclusion:**

les urgences urologiques sont dominées dans notre contexte par la rétention d´urine vésicale en rapport avec les affections prostatiques et de rétrécissement de l´urètre. Les urgences traumatiques sont peu fréquentes.

## Abstract (en)

**Introduction:**

urological emergencies are diverse and varied, and are frequently encountered in urological practices. The purpose of this work was to study the epidemiological features and management of urological emergencies at the regional hospital of Kolda, Senegal.

**Methods:**

we conducted a retrospective and descriptive study collecting data from the medical records of all patients admitted as a urological emergency for 20 months (January 2020 and August 31, 2021).

**Results:**

urological emergencies accounted for 3.6% of admissions to the Emergency Department and for 20.4% of urological consultations. The average age of patients was 51.9 ± 21.5 years, ranging from 1 to 98 years. Urinary retention was the most common urological emergency, observed in 249 cases (57.3%), followed by urogenital infections 89 (20.5%). Renal colic affected 48 (11%) of patients. Emergency management was based on urinary catheterization, which was performed in 193 patients (44.3%). The most common surgical procedures were suprapubic catheterization (42 patients; 51.9%) and emergency scrotal exploration (15 patients; 18.6%).

**Conclusion:**

in our context, urological emergencies are dominated by urinary retention due to prostatic disorders and urethral stenosis. Traumatic emergencies are uncommon.

## Introduction

L´urologie est une spécialité médico-chirurgicale, qui connait un nombre important de situations nécessitant une prise en charge en urgence afin d´éviter des séquelles graves, des complications voire le décès du patient. Les urgences urologiques généralement rencontrées dans les services des urgences sont nombreuses et variées. Il peut s´agir des affections scrotales aiguës, du priapisme, de la rétention d´urine vésicale aiguë ou chronique, de la colique néphrétique, de l´hématurie macroscopique, des infections urinaires, des traumatismes génito-urinaires, etc. Dans les séries occidentales [1], les lombalgies occupent la première place des consultations en urgence, alors que dans les séries africaines, la rétention d´urine vésicale constitue le premier motif d´admission aux urgences [2-5]. Les services des urgences sont des unités importantes au sein d´un hôpital où les soins sont dispensés aux patients nécessitant une prise en charge en urgence. Les équipes de garde sont donc régulièrement confrontées à la prise en charge de ces urgences au niveau du service d´accueil des urgences. Au Sénégal, quelques études sur ce sujet réalisées en milieu rural sont disponibles [5,6]. Aucune donnée épidémiologique sur les urgences urologiques sont disponibles dans cette région du sud du Sénégal. L´objectif de ce travail était d´étudier les aspects épidémiologiques et la prise en charge thérapeutique de ces patients au centre hospitalier régional de Kolda, Sénégal.

## Méthodes

**Cadre de l´étude:** l´hôpital régional de Kolda, situé au sud du Sénégal, est un hôpital de niveau 2. Par sa situation géographique, c´est un hôpital à vocation sous régionale. On y reçoit des patients de la région, et ceux venus de la Gambie, de la République de Guinée et de la Guinée-Bissau.

**Type de l´étude:** nous avons réalisé une étude rétrospective et descriptive portant sur une population de 435 patients durant la période de l´étude (1^er^ janvier 2020 au 31 août 2021) soit 20 mois. Nous avons procédé à un échantillonnage exhaustif des dossiers de tous les patients reçus pour urgence urologique. Les urgences urologiques étaient reçues au SAU (Service d´Accueil des Urgences) tenu par un médecin généraliste et une équipe de deux infirmières; puis le patient était ensuite orienté à l´urologue pour la suite de prise en charge. Cependant, d´autres patients étaient directement reçus en consultation urologique.

Nous avons inclus dans notre étude, tous les patients qui ont été reçu pour une urgence urologique au niveau du service d´accueil des urgences ou en consultation urologique durant la période de l´étude et qui ont été effectivement pris en charge. Les patients dont les urgences ont été levées au niveau des centres de santé et les patients reçus pour des urgences extra-urologiques (hernie étranglée et autres urgences digestives) ont été exclus.

Les données ont été recueillies à partir des registres du service d´accueil des urgences, des registres de consultations urologiques, des registres d´hospitalisation et de compte rendu opératoire. Les paramètres étudiés étaient l´âge, le sexe, la fréquence, les motifs d´admission, les gestes effectués en urgence (sondage vésical ou acte chirurgical), le délai d´hospitalisation au SAU, ainsi que les étiologies de la rétention d´urine vésicale.

**L´analyse statistique:** les données ont été saisies et analysées à l´aide du logiciel Epi-info 7.1.1.1. Les variables quantitatives (âge) ont été exprimées en moyenne ± écart-type et les variables qualitatives en pourcentage et effectifs.

**Considérations éthiques:** l´étude a été approuvée par la Commission Médicale d´Établissement, qui regroupe les médecins, chefs de service de notre institution. Cette commission faite fonction de comité éthique. Les règles d´anonymat et de confidentialité ont été respectées selon la déclaration d´Helsinki.

## Résultats

**Figure 1:**
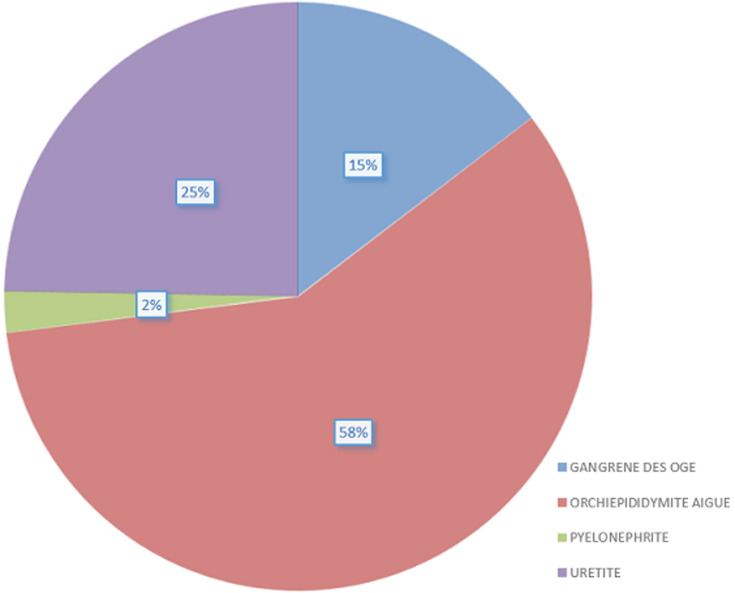
principales étiologies des infections uro-génitales

Durant la période de l´étude, les patients admis pour urgence urologique représentaient 3,6% (435/12253) de toutes les urgences admises au Service d´Accueil des Urgences (SAU) d´une part, et d´autre part, les urgences urologiques représentaient 20,4% des consultations urologiques (435/ 2135). L´âge moyen des patients était de 51,9 ± 21,5 ans avec des extrêmes de 1 an et de 98 ans. Plus de la moitié des patients (51%) avaient un âge supérieur ou égal à 61 ans. Les urgences urologiques concernaient essentiellement les hommes, soit un sexe ratio de 18,7. La rétention d´urine vésicale a été l´urgence urologique la plus fréquente 249 cas (57,3%), suivie des infections urogénitales 89 cas (20,5%). Le Tableau 1 répertorie les urgences urologiques reçues. Parmi les 249 patients qui présentaient de la rétention d´urine vésicale, l´hypertrophie bénigne de la prostate (HBP), la sténose de l´urètre et le cancer de la prostate ont été les principales étiologies retrouvées (Tableau 2). Les infections uro-génitales retrouvées étaient les orchiépididymites aiguës (OEA), les urétrites et la gangrène des organes génitaux externes (OGE) comme le montre la Figure 1. La colique néphrétique avait concerné 48 cas (11%), dont 39 cas étaient en rapport avec un calcul urinaire du haut appareil. L´hématurie sans caillot vésical et la torsion du cordon spermatique (TCS) représentaient respectivement 4,4% et 2,5% des urgences urologiques. Les urgences traumatiques concernaient essentiellement le bas appareil urinaire. Il s´agissait de 3 cas de fracture de la verge et 6 cas de traumatismes des bourses dont 4 cas avaient nécessité une exploration chirurgicale en urgence.

**Tableau 1:** répartition des patients selon le type d´urgence urologique

Urgences urologiques	Effectif	%
Anurie obstructive	1	0,2
Colique néphrétique	48	11
Hématurie sans caillot	19	4,4
Infections	89	20,5
Phimosis	1	0,2
Priapisme	7	1,6
Prostatite aigue	1	0,2
Rétention d´urine vésicale	249	57,3
Torsion du cordon spermatique	11	2,5
Traumatismes des OGE (GBA + fracture de la verge)	9	2,1
**Total**	**435**	**100**

**Tableau 2:** répartition des patients selon les étiologies de la rétention d´urine vésicale

Les étiologies de la rétention d´urine vésicale	Effectif	%
Rétention d´urine du post-partum	2	0,8
Phimosis	1	0,4
Sténose de l'urètre	55	22,1
Hypertrophie bénigne de la prostate (HBP)	173	69,5
Cancer de la prostate	15	6,0
Rétention d´urine par caillot vésical	3	1,2
**Total**	**249**	**100**

Pour la prise en charge des urgences, le sondage vésical transurétral a été réalisé chez 193 patients (44,3%) dont 3 cas ayant nécessité un décaillotage et irrigation vésicale. Les coliques néphrétiques ont été traitées médicalement par l´association d´anti-inflammatoire non stéroïdien et d´antalgique. Le patient ayant présenté une anurie obstructive a été orienté pour une montée de sonde double J dans un centre disposant de l´endo-urologie. Le passage aux urgences a été de courte durée (moins de 24 heures) pour la majorité des patients et le nombre d´hospitalisations en urgence était de 109 (25,1%) patients. L´orchidectomie pour TCS négligée était faite chez 5 (45,5%) patients ayant eu une torsion du cordon spermatique (n=11). Les actes chirurgicaux réalisés en urgence étaient dominés par le cathétérisme sus-pubien et l´exploration en urgence du scrotum pour grosse bourse aigue (GBA) (Tableau 3).

**Tableau 3:** les actes chirurgicaux réalisés en urgence

Gestes réalisés en urgence	Effectif (n= 80)	%
Albuginorraphie	3	3,7
Anastomose caverno-spongieuse	3	3,7
Cathétérisme sus-pubien + débridement	13	16,0
Cathétérisme sus-pubien	42	51,9
Ponction des corps caverneux	4	4,9
Exploration Scrotale (TCS + GBA post-traumatique)	15	18,6
Circoncision	1	1,2
**Total**	**81**	**100**

## Discussion

L´admission dans un contexte d´urgence est le mode habituel de découverte des pathologies urologiques dans les hôpitaux en Afrique. Bien que les urgences urologiques soient moins fréquentes par rapport aux urgences des disciplines médicales, elles occupent une part importante des activités dans un service d´accueil des urgences. Les urgences urologiques représentaient en moyenne 21 urgences par mois. Ce résultat supérieur à celui de Diabaté *et al*. [5] qui rapportaient en moyenne 11,74 urgences par mois. Cependant, ce résultat est proche de celui trouvé par Boissier R *et al*. [7] en France, qui rapportaient que les urgences urologiques représentaient en moyenne 4,2% de la totalité des passages aux urgences. L´âge moyen de nos patients était de 51,9 ± 21,5 ans. Ce résultat montre que les urgences urologiques concernent essentiellement les sujets âgés. Des auteurs expliquent cela par le fait que les pathologies prostatiques surviennent à partir de la cinquième décade, et que leur fréquence augmente avec l´âge [5]. En Guinée et au Sénégal, les patients de 60 ans et plus admis en urgence représentaient respectivement 58% et 50,7% des cas [2, 3]. La plupart des auteurs [1-5, 8], comme dans notre étude, retrouvent une prédominance masculine. Cette prédominance masculine est liée à la fréquence plus élevée dans notre contexte des urgences liées aux pathologies urétro-prostatiques.

Sur le plan clinique, la rétention d´urine vésicale a été l´urgence urologique la plus rencontrée. Elle constitue la principale urgence urologique dans les séries africaines [2-5, 8]. Cela s´explique par des raisons socio-culturelles. En effet, dans nos sociétés, le dépistage des maladies étant rare, les malades ne consultent qu´au stade des complications [3, 6]. Les étiologies les plus fréquentes de la rétention d´urine dans cette étude étaient dominées par les affections urétroprostatiques. Ce résultat est similaire à ceux d´autres études réalisées en Afrique [2, 3, 8, 9]. Diallo AB *et al*. expliquent [2] cela par le taux d´analphabétisme parmi ses patients et par le fait que les malades commencent le plus souvent chez le guérisseur traditionnel avant de consulter à l´hôpital, comme c´est le cas de nos patients. Cette incidence élevée de la rétention d´urine vésicale a pour conséquence un port prolongé de la sonde vésicale en attente de chirurgie. Cela peut engendrer des complications infectieuses, notamment les OEA.

Dans les pays développés, Mondet *et al*. [1] en France rapportaient que la symptomatologie douloureuse lombaire était le motif de consultation le plus fréquent et que la rétention d´urine et la dysurie représentaient respectivement 15,2%, et 34,7% des consultations chez ses patients. Boissier R *et al*. [7] quant à eux notaient, une prédominance de la colique néphrétique suivie de la rétention d´urine vésicale et l´hématurie chez les hommes et que la cystite et la colique néphrétique étaient prédominantes chez les femmes. L'infection du système urinaire est un problème de santé rencontré partout à travers le monde [10]. Nous avons noté dans cette étude que les pathologies infectieuses étaient fréquentes. Elles sont dominées par les orchiépididymites, suivies des urétrites. Certains auteurs ont rapporté des résultats similaires. Tengue K [11] rapportait 22,2% des infections avec une prédominance des OEA, suivie de la gangrène de fournier. Diabaté I [5] soulignait déjà la diversité de ces infections et rapportait la prédominance des orchiépididymites comme ce fut le cas dans notre série. Le constat comme quoi, la fréquence des gangrènes des OGE et la rareté des pyélonéphrites était déjà rapporté dans les études au Sénégal et au Togo [3, 11] comme dans cette série. Les gangrènes des OGE sont le plus souvent la circonstance de diagnostic du rétrécissement au stade de complication. Le traitement de ces infections uro-génitales était médical dans les orchiépididymites aiguë ou chirurgicale par un débridement en cas des gangrènes des OGE associé à un drainage des urines par un cathétérisme vésical sus pubien. Une tri-antibiothérapie associant une céphalosporine, le flagyl et la gentamycine était instituée pour le traitement post-opératoire. Ces modalités de traitement ont été les mêmes chez d´autres auteurs [2, 3, 5]. Contrairement aux auteurs africains, Martin L [12] en France notait une fréquence de 27% des pyélonéphrites obstructives et que le drainage en urgence des pyélonéphrites représentait 31% des interventions effectuées.

Contrairement à certaines études menées en Afrique [2, 11] où la fréquence de la colique néphrétique reste basse, 4,2% et 2,3% respectivement en Guinée et au Togo; dans notre série, la colique néphrétique représentait la 3^e^ étiologie des urgences urologiques reçues dans notre structure et avait pour origine un calcul urinaire chez 39 patients, soit 81,3% des cas de colique néphrétique. Diamé ID [7] rapportait des résultats similaires. Par ailleurs, en France, Martin L [12] rapportait que la colique néphrétique était la deuxième cause de consultation en urgence. Le facteur environnemental est déterminant dans la lithogenèse. Ceci pourrait expliquer l´incidence élevée des calculs urinaires dans les régions tropicales à l´origine de la colique néphrétique, comme c´est le cas dans notre série.

La torsion du cordon spermatique était peu fréquente dans notre étude. Fall et Tfeil [4, 8] rapportaient des résultats similaires avec respectivement 2,8% et 2,9%. Par ailleurs, des résultats similaires sur la fréquence de la torsion du cordon spermatique ont été rapportés par Talreja S *et al*. [13] qui avaient trouvé 2,04%. Cependant, Traoré *et al*. [14] rapportaient une fréquence plus élevée de TCS, 4,2%. Du fait de retard de consultation pouvant aller jusqu´à une semaine comme nous avons pu le constater dans cette série, le taux d´orchidectomie était élevé. Ce résultat est similaire à celui de Owon´Abessolo PF [4] qui rapportait un taux d´orchidectomie de 41,2%. La TCS est une urgence qui doit être connue par tous les agents de santé. Lorsqu'une torsion testiculaire est suspectée, une exploration chirurgicale immédiate est obligatoire afin de réduire le risque d´orchidectomie.

La proportion des traumatismes urogénitaux rencontrés dans cette étude était faible. La fracture du pénis et lésions scrotales sont relativement rares [15] Nous avons observé comme Fall et Diabaté [3, 5] des traumatismes portant essentiellement sur les OGE. Il s´agissait de cas de fracture de la verge par faux pas, de coït réparé chirurgicalement et de GBA post-traumatique survenue essentiellement lors de pratique de sport. Une scrototomie d´exploration pour hématocèle et une suture de l´albuginée pour fracture testiculaire ont été effectuées.

**Limites:** la présente étude présentait certaines limites, du fait de son caractère rétrospectif, le devenir des malades à court et à moyens termes n´a pas été évalué.

## Conclusion

Les urgences urologiques sont fréquentes dans notre pratique quotidienne. Elles sont dominées dans notre région par la rétention d´urine vésicale en rapport avec les affections prostatiques et de rétrécissement de l´urètre. Elles concernent majoritairement les hommes. Les urgences traumatiques portent essentiellement sur le bas appareil urinaire, et sont peu fréquentes.

### Etat des connaissances sur le sujet

- Les urgences urologiques sont fréquentes dans la pratique urologique au quotidien;
- En Afrique, la rétention urinaire occupe la première place des urgences urologiques;
- La rareté des gangrènes des OGE dans les séries occidentales.

### Contribution de notre étude à la connaissance

- Le retard en milieu rural de consultions des cas de torsion de cordon spermatique, aboutissant à des proportions élevées d´orchidectomie;
- La prédominance des orchiépididymites dans les infections urogénitales et la rareté des cystites;
- La rareté des traumatismes des voies urinaires dans les urgences urologiques et notamment l´absence de traumatisme du haut appareil urinaire.
